# Evaluation of the Efficacy of Artificial Neural Network-Based Music Therapy for Depression

**DOI:** 10.1155/2022/9208607

**Published:** 2022-08-21

**Authors:** Qian Ding

**Affiliations:** College of International Exchange, Shandong Management University, Jinan 250357, China

## Abstract

In order to evaluate the therapeutic effect of music therapy on patients with depression, this paper proposes a CNN-based noise detection method with the combination of HHT and FastICA for noise removal, with good data support from the DBN model. DBN-based feature extraction and classification are completed. As the training process of DBN itself requires a large number of training samples, there are also disadvantages such as slow convergence speed and easy to fall into local minima, which lead to a large amount of effort and time, and the learning efficiency is relatively low. A DBN optimization algorithm based on artificial neural network was proposed to evaluate the efficacy of music therapy. First of all, through the comparison of music therapy experimental group and control group, to verify that music therapy is effective for the treatment of depressed patients. Secondly, we propose to optimize the selection of features based on the frequency band energy ratio and the sliding average sample entropy, respectively, and then to classify the EEG of depressed patients under different music perceptions by training the DBN model and continuously adjusting the parameters, combined with the surtax classifier, and the classification accuracy is high. In particular, it can detect the different effects of different music styles, which is of great significance for the selection of appropriate music for the treatment of depressed patients.

## 1. Introduction

Production of abnormal sound sensations in the absence of any external stimuli is known as tinnitus. It is a symptom of an auditory disorder in which depressed patients perceive a voice in their head or ears, but there is no sound source in the actual environment [1]. The prevalence of tinnitus is as high as 15% to 20% and it is estimated that close to 200 million people suffer from tinnitus [2]. It tends to increase with age. Tinnitus is often associated with deafness, but it is more distressing than deafness [3] and can cause serious psychological disturbances and even suicide. Tinnitus is a subjective sensation and there are no objective tests. Nor are there any specific medications or treatments available. Tinnitus is a pressing medical problem in clinical practice. In recent years, a large amount of literature has been published on the diagnosis and treatment of tinnitus in China, involving dozens of treatment methods, such as music therapy, masking therapy, and cognitive therapy [4], especially Chinese medicine treatment methods are more numerous [5], mainly focusing on vasodilation, improving microcirculation, reducing blood viscosity, neurotropism, etc. The efficacy is not ideal [6].

While people continue to be familiar with the relationship between medicine and music, there is also a greater awareness of their scientific principles, and even more so, music perception therapy has been put into practice. The modern clinical treatment of music perception in China began in the late 1970s [7]. With the rapid development of music perception therapy, clinical music perception therapy for children's depression has also been gradually developed. Studies have shown that music perception is the treatment of choice for improving the attentional and social skills of depressed patients, as well as improving physical coordination [8]. Numerous studies have shown that the brain's level of arousal when listening to music is mainly in the right brain, which also controls much of a person's emotions and behaviour, as well as improving the behavioural abilities of children with intellectual disabilities, and its application in special education and the education of depressed patients is a mainstream trend [9, 10].

EEG and mental disorders are closely linked, and numerous studies have shown that EEG waves have characteristic abnormalities such as amplitude, power, and left-right asymmetry that is different for depressed patients than in healthy people. This will have significant implications for the prevention of mental disorders as well as their diagnostic treatment [11]. The clinical process of analysing EEG signals from depressed patients is limited by the diagnostic skills of physicians, and manual diagnosis is often slow and subjective, so more and more researchers are focusing on deep learning algorithms and applying them to the automatic identification and classification of EEG signals [12]. Deep learning has made significant breakthroughs in the field of machine learning, particularly in the application of speech signal recognition as well as image recognition, which makes it of great relevance for EEG classification problems. However, not all problems can be solved with good results using deep learning, for example, for natural language processing and logical reasoning, which can not be effectively solved by deep learning.

Whether music perception therapy has positive effects on depressed patients and how to effectively apply deep learning to the classification of EEG in depressed patients, to improve the accuracy of EEG classification and to determine the condition accurately, is a very relevant study.

## 2. Related Works

Music is inextricably linked to many human activities and is of great importance to humans because of the emotional feedback it possesses. Music triggers electrophysiological activity in the brain and expresses musical emotions in the form of brain waves [13]. There was no significant difference between the two groups in terms of scores, while there was a positive correlation between auditory performance and melody discrimination. With regard to the treatment of depressed patients, music is one of the indispensable options. This brings us to a recent research, hotspot-music perception therapy [14].

Music can stimulate the release of certain transmitters that affect brain function (e.g., norepinephrine), thus promoting the coordination of function in various areas of the brain. This is because the centres that primarily control emotions in the human brain are the hypothalamus and the limbic system, and music, by acting directly on these areas, can regulate emotions in both directions, and therefore can also help to treat certain physical and mental disorders [15]. The temporal lobe region of the brain contains both the auditory central system and the nociceptive central system of the brain, and since musical stimulation makes the auditory centre work more actively and thus has an inhibitory effect on pain, music can alleviate pain to some extent. Brain areas that dominate musical activity are mainly in the right hemisphere of the brain. Interventions in music perception can improve brain function, harmonise the balance between the left and right hemispheres of the brain, thereby accelerating intellectual development, as well as improving the cognitive abilities of children with intellectual disabilities and being used in special education and the education of depressed patients. In clinical practice music perception therapy is mainly used in pain interventions, treatment of depressed patients, psychotherapy for children, dementia, psychosis, speech disorders, emotional disorders, cancer, hospice care, etc., [16].

It is confirmed that music can be used as a source of emotional cognitive stimulation in the process of exploring the brain's mechanisms of action, an idea that can be justified in biomedical terms [17]. In order to capture the ERP produced by the human brain, brief sounds were used as a source of arousal, and the site of action of the brain in processing music was studied by analysing changes in the characteristics of the EEG signal during the experiment, and the right half of the temporal lobe was found to be significant [18]. Introducing music features into the study of music therapy, proposed an SVM-based model of emotional perception of music, and analysed the effects of heart rate, electrical skin conductivity, respiratory rate, blood pressure, and body temperature on the perception of music from the perspective of the physiological effects of music [19]. In the literature of emotion model research based on brain wave music, the emotional information contained in brain wave signals is explained in the form of music [20].

It is confirmed that musical activities were particularly effective in improving verbal communication skills in individual cases [21]. Investigated the characteristics of specific biomarkers on the brains of depressed patients and analysed changes in EEG signals through interventional treatment of marked brain areas [22].

## 3. EEG Signal Acquisition for Music Perception

EEG signal acquisition is a necessary part of the analysis of EEG signal characteristics and subsequent processing and application, and there are two main types of EEG data acquisition methods: implacable and nonimplantable [23].

### 3.1. Screening of Experimental Subjects

A total of 28 depressed patients were screened, taking into account the potential pitfalls of intellectual limitations or hyperactive behaviour that depressed patients may have during the experiment. Each subject's musical experience and musical needs were investigated and counted using questionnaires, interviews, and literature analysis. Each subject's favourite songs, most frequently listened to songs (e.g., children's sleep songs), disliked songs, and unfamiliar song styles were counted and recorded. With reference to these records and the medical approach to the selection of musical pieces during music therapy for depressed patients, four types of music were finally identified: (1) dirge, (2) unfamiliar symphonic music, (3) upbeat music, and (4) unfamiliar bland music, plus no music experimental setting for comparison were set.

### 3.2. Music Selections

For music perception therapy to have a reassuring effect on depressed patients, the first step is to choose the right music. Music is an art of sound and not all music is suitable for depressed patients to use for healing. Firstly, the music should not span too wide a range, but also be gentle and not have extreme pitch changes that sound discordant. Secondly, each subject is an individual and their sensitivity to musical styles varies greatly, so it is important to select music specifically for each subject while meeting the popular criteria for treating depression [24].

## 4. Optimal Selection of Feature Data

We know that DBN's EEG signal recognition method allows the computer to automatically extract from the input data for the low-level and high-level features that characterise these sample signals in an unsupervised manner, resulting in better classification recognition performance. Finding feature data that characterise the differences between the EEG of depressed patients and that of healthy children is therefore a priority.

As the distribution of individual rhythms in the EEG of clinically diagnosed depressed patients can be highly abnormal of values that can be used to match the frequency bands of the individual EEG rhythms [25]. So a harmonic wavelet packet transform is first performed. Using the transformed wavelet coefficients *hwpt*(*s*, *i*, *k*) the bond energy of each rhythm is calculated using the following equation:

(1) $$\begin{array}{c} E^{\left(p\right)}\left(k\right)=\sum_{i}{\left|hwpt\left(\left(s,i,k\right)\right)\right|}^{2}. \end{array}$$

The range of *k*=0,1,…, *N* − 1; *p*=*δ*, *θ*, *α*, *β*,….*i* is determined by the frequency band of the rhythm represented by *p*. The FBER for each rhythm in the lead corresponding to a given electrode patch is calculated by the following equation:

(2) $$\begin{array}{c} E_{\text{all}}\left(k\right)=\sum_{p}E^{\left(p\right)}\left(k\right), \end{array}$$

(3) $$\begin{array}{c} \text{FBER}-S=\frac{E^{\left(p\right)}\left(k\right)}{E_{\text{all}}\left(k\right)}\times 100\%. \end{array}$$

## 5. DBN Model Design

Deep learning networks are derived from artificial neural networks. The vast majority of current machine learning algorithms are shallow in nature and are constrained in their ability to generalise to complex classification problems. EEG signals are nonlinear, nonstationary, and very weak, and surface-level time-domain features largely fail to distinguish some pathological EEG signals, while deep belief networks provide a measure of multilayer learning of features.

**Figure 1:**
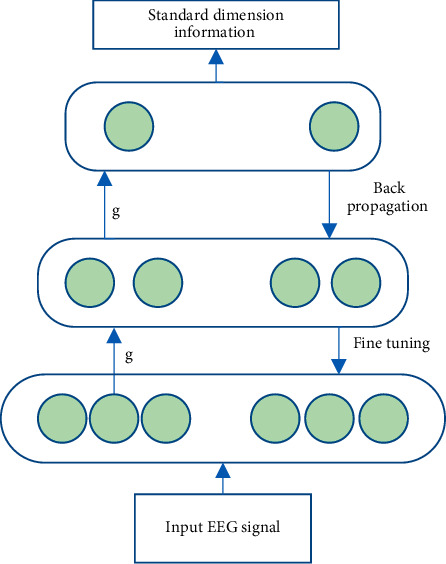
DBN network architecture.

A typical deep belief network DBN structure and working principle is shown in Figure 1.

The mathematical model of DBN can be represented in the following form:

(4) $$\begin{array}{c} P\left(x,g^{\left(1\right)},g^{\left(2\right)},\ldots ,g^{\left(l\right)}\right)=P\left(x|g^{\left(1\right)}\right)P\left(g^{\left(1\right)}|g^{\left(2\right)}\right)\cdots P\left(g^{\left(l-2\right)}|g^{\left(l-1\right)}\right)P\left(g^{\left(l-1\right)}|g^{\left(1\right)}\right), \end{array}$$

where *x*=*g*^(0)^, *g*^(*l*)^ represents the *l*-th level weight matrix.

The softmax regression model differs from the binary classification model in that it is a generalisation of the logistic regression model to a multiclassification problem, which is more suitable for classifying multilabelled data [26].

In softmax regression, the hypothesis function outputs a *k*-dimensional vector (the sum of each vector element is the probability value of these *k* estimates). For a given test input sample (*x*, *y*), the hypothesis function is used here to calculate the probability value *p*(*y* = *j* | *x*) corresponding to each category *j*. Our hypothesis function *h*(*x*) takes the following form:

(5) $$\begin{array}{c} h_{\theta }\left(x^{\left(i\right)}\right)=\left[\begin{array}{c} p\left(y^{\left(i\right)}=1|x^{\left(i\right)};\theta \right)\\ \vdots \\ p\left(y^{\left(i\right)}=2|x^{\left(i\right)};\theta \right)\\ p\left(y^{\left(i\right)}=k|x^{\left(i\right)};\theta \right) \end{array}\right]=\frac{1}{\sum_{j=1}^{k}e^{\theta_{j}^{\theta }x^{\left(x\right)}}}\left[\begin{array}{c} e^{\theta_{1}^{T}\left(i\right)}\\ e^{\theta_{2}^{T}x^{\left(i\right)}}\\ e^{\theta_{k}^{T}x^{\left(i\right)}} \end{array}\right], \end{array}$$

where *θ*_1_, *θ*_2_,…, *θ*_*k*_ ∈ *ℜ*^*n*+1^ is a parameter of the model.

In terms of the mathematical model, the softmax cost function is particularly similar to the logistic regression cost function, with the slight difference that in the loss function of softmax, those *k* probability values for each class marker are cumulated [27]. The probability of classifying *x* as class *j* in a softmax regression is as follows:

(6) $$\begin{array}{c} p\left(y^{\left(i\right)}=j|x^{\left(i\right)};\theta \right)=\frac{e^{\theta_{j}^{T}x^{\left(i\right)}}}{\sum_{i=1}^{k}e^{\theta_{i}^{T}x^{\left(i\right)}}}. \end{array}$$

A shallow artificial neural network based on the softmax regression model, which mainly uses the gradient descent formula of softmax to correct the neuron weights, using the softmax regression model can support the statistical significance of the classifier in mathematical theory.

## 6. Experiment

At present, a large number of studies have proved the value and effectiveness of music therapy in the treatment of patients with depression. Before testing the evaluation efficiency of DBN Model, a control analysis was conducted on the efficacy of music therapy.

**Figure 2:**
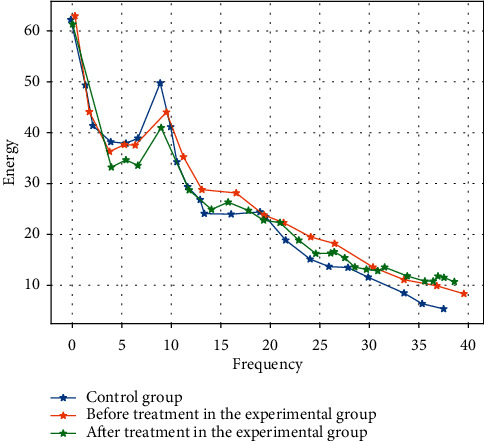
Graph of experimental group before and after treatment and control group.

There was statistically significant difference in EEG between the experimental group and the control group before treatment (*t* = 4.404, *P* < 0.05). There were statistically significant differences in the full-band energy of frontal, parietal, temporal, and occipital of 28 people with depression before and after treatment (*t* = 3.705, *P* < 0.05). The comparison of *δ*, *θ*, *α*, and *β* band energy in EEG of autistic children before treatment was significantly lower than that of after treatment, and also lower than that in control group. The comparison of EEG *γ*-band energy of people with depression before treatment was slightly higher than that of after treatment and also slightly higher than that of the control group. After treatment, the energy of brain regions in the experimental group and the control group was roughly the same, with no statistical significance (*P* > 0.05, Figure 2). It shows that the EEG of people with depression restored to the normal individual level after music therapy, and the curative effect is very obvious.

In the most important step of EEG classification, the feature data matrix extracted in the previous section is used as input data to the software classifier model. In this case, each row of the feature matrix is obtained by superimposing 25 sliding sample entropy values and 25 frequency energy scale values. The number of columns is a predetermined list of samples of EEG signals from depressed patients in five settings, with each patient feature represented by 208 samples, giving a total of 1456 rows. Of these, 150 were used as the training set and 58 as the test set.

**Figure 3:**
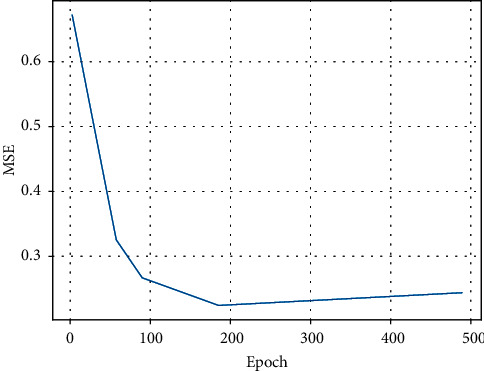
Mean square error decline in iterations.

Sophia is a typical multiclassifier, where the output is a *k*-dimensional vector, where each value represents the probability of the classification, and the sum of the elements of the vector is 1. The experiment was trained and tested for 35 iterations, with each iteration reshuffling the feature data samples. The gradient descent algorithm was used to adjust the parameters to minimise the error.  Figure 3 below shows the mean squared error (MSE) of the classifier as the number of iterations increases, with 500 iterations of the process.

Overall, the MSE decreases as the number of iterations increases, and after 80 iterations the mean square error has stabilised.

When the training set was trained by the model to build a model with minimal error, the process was unsupervised, and then before the test set was fed into the model, we labelled the data in each environment, five in total, the process was supervised, this was to facilitate the observation of where the EEG data in each environment went after classification to build a confusion matrix to analyse the perceptual effect of each music, the higher the accuracy of the genre, the greater the effect on the brain waves. Ultimately, the model outputs a series of numbers representing the classification accuracy.

**Figure 4:**
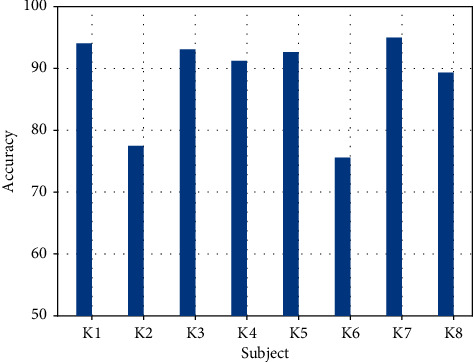
Histogram of classification accuracy for 28 depressed patients.

Figure 4 below shows the results of the classification statistics for each of the 28 people with depression. As showed by the bar graphs, it was found that there were still differences in classification accuracy between the different depressed patients, which indicate the variability of musical sensitivity to different people. The classification accuracy of the EEG of most of the subjects ranged from 75% to 94%, with a maximum of 94.07% and a minimum of 76.24%, with an average accuracy of 84.78%, which also demonstrates the stability of the DBN classification model.

Table 1 is a confusion matrix of the results of the DBN's classification of EEG in five environments for depressed patients. The data therein represent the proportion of EEG in each musical style that was classified into each of the five environments, and the proportion that was classified into itself represents the accuracy of the EEG in response to such musical perception.

From this, it was found that the classification accuracy was higher in the Music 1 and Music 3 environments. This suggests that the EEG evoked in these two environments is more easily distinguishable from the EEG characteristics of the other environments, i.e., Music 1 and 3 have a greater effect on the person's condition (EEG waveform), either alleviating the autistic mood and making the person more positive, or causing the condition to worsen and become more negative. Conversely, Music 2 and Music 4 was classified less accurately, suggesting that the music in these two environments had little effect on the condition, resulting in a poor differentiation of the EEG signal characteristics.

In order to analyse whether Music 1 and Music 3 had a positive or negative effect on the EEG, transient frequencies of EEG under Music 1 and Music 3 were obtained. By simulating the frequency characteristics of each segment of the EEG, we compared the frequency characteristics of the EEG of Music 1 and Music 3 with those of the EEG in the no-music environment, and found that the frequency characteristics of the EEG of Music 3 were basically higher than those of the no-music environment, while the frequency characteristics of the EEG of Music 1 were lower than those of the no-music environment. This suggests that music 3 had a more alleviating effect on the depressed patient's condition than if he had not listened to music, while Music 1 exacerbated the condition, as evidenced by the lower frequency profile and the more depressed mood of the patient.

## 7. Conclusions

In this paper, an optimised DBN feature extraction and classification method are proposed in order to more accurately classify the EEG of depressed patients under the effect of different music perceptions. By introducing the structure and working principle of typical deep trust network DBN, and selecting HHT and FastICA, the optimized DBN feature extraction is completed. The optimised features can better characterise the deeper information of the original signal than the traditional course data set as input data. Finally, the surtax method is used to classify the extracted features. The experimental results show that the model is optimally trained, which paves the way for subsequent analysis of the classification accuracy. The optimised DBN algorithm can evaluate the efficacy of different music styles for depressed patients, which has direct guiding value for the effective selection and creation of music in the treatment process.

## Figures and Tables

**Table 1:** Confusion matrix for classification of depressed patients' outcomes.

People with depression/%	No music 0	Music 1	Music 2	Music 3	Music 4
No music 0	20.37	31.49	19.2	12.77	16.17
Music 1	1.28	88.65	3.14	5.11	1.82
Music 2	38.24	12.46	31.21	3.84	14.25
Music 3	2.87	4.36	1.92	85.26	5.59
Music 4	27.16	18.59	10.12	18.44	25.33
Accuracy	20.37	88.65	31.21	85.26	23.33

## Data Availability

All data generated or analysed during this study are included in this article.
